# Location Choice in the Context of Older Adults’ Leisure-Time Walking

**DOI:** 10.3390/ijerph17134775

**Published:** 2020-07-02

**Authors:** Zhengying Liu, Astrid Kemperman, Harry Timmermans

**Affiliations:** Urban Planning and Transportation Group, Eindhoven University of Technology, 5600 MB Eindhoven, The Netherlands; a.d.a.m.kemperman@tue.nl (A.K.); H.J.P.Timmermans@tue.nl (H.T.)

**Keywords:** location choice, leisure-time walking, older adults, open space, neighborhood environment

## Abstract

Leisure-time walking is the most prevalent and preferred form of physical activity of older adults. In order to promote leisure-time walking and enhance the efficiency of using outdoor open spaces, the supply of different types of walking locations should match the needs, interests and preferences of older adults. However, there is limited knowledge on which location types are chosen by which groups of individuals under which conditions. This study therefore examines the effects of socio-demographics, episode participation attributes and neighborhood characteristics on the location choice of older adults for leisure-time walking. A multinomial logit model is estimated based on data collected among 316 respondents aged 60 or older in Dalian, China. The results indicate that older people’s location choices for walking are associated with their socio-demographics, episode participation attributes and neighborhood characteristics. Finally, implications of the results for the planning, design and management of open spaces are identified.

## 1. Introduction

The population of older adults has been increasing all over the world and many of them suffer from chronic diseases [1,2]. An increasing body of research in the public health field suggests that regular physical activity in old age prevents the onset and development of chronic diseases and helps to maintain functional independence and good quality of life [3].

Parks that provide low- or no-cost places for physical activity have drawn increasing attention to its effect on physical activity. This is reflected in the exponential growth over recent years of studies examining the relationships between physical activity and park characteristics [4,5,6,7,8,9]. These studies have found that attributes of parks can positively influence people’s physical activity levels. For example, closer proximity to parks is associated with higher levels of physical activity among older adults [5]. Thus, investment in parks may be an effective way to boost older adults’ physical activity opportunities and thereby produce health benefits.

Neighborhood streets also provide residents with important opportunities for much of their physical activity. Previous studies have shown that neighborhood streets are the setting most frequently used for recreational physical activities [10,11]. The small but growing body of research has documented the positive effects of street characteristics on physical activity. For example, Sugiyama et al. [12] found that attractiveness (lots of greenery, free from litter rubbish and graffiti, etc.) is significantly associated with the frequency of using neighborhood streets for recreational activity or exercise among Australian adults. Giehl et al. [13] found a positive association between a higher percentage of sidewalks and walking for transportation of older adults in Brazil. Thus, investment in streets may be another effective strategy to increase street-based physical activity.

However, in practice, the socioeconomic disparities in park accessibility and the disparities in the provision of parks are hard to avoid. This means that investments in parks such as adding park facilities would only benefit those who have better access to parks. Moreover, some individuals may prefer parks for physical activity while others may prefer other places such as streets for physical activity. If this were the case, investments in parks would only benefit those who use parks for physical activity. The other intervention strategy, investments in streets might also only benefit those who prefer streets for physical activity.

An intervention that could benefit greater numbers of people is better and should be given a priority for implementation. We argue that if most people prefer streets to parks for physical activity due to the effects of their socio-demographic characteristics and activity habits in terms of activity time and activity duration, etc., investments in streets would be a better intervention and vice versa. However, there is currently a dearth of information concerning empirical relationships between socio-demographic characteristics, contextual features of activity participation and location choice for physical activity. Without such evidence, it will be difficult for decision makers to evaluate the cost effectiveness of different interventions designed for promoting physical activity.

Moreover, in order to enhance healthy aging through promoting physical activity among older adults, it is crucial to match the supply of physical activity location types to the needs, interests and preferences of older adults. Nevertheless, creating environments that accommodate the various preferences of physical activity participants is challenging for urban planners and designers, because knowledge about the diversity of older adults’ preferences for different physical activity location types and the relationship between these preferences and their socio-demographic characteristics and neighborhood characteristics is rather limited.

To address these gaps, we investigated what the effects of contextual features of activity participation, socio-demographics and neighborhood characteristics on location choice for physical activity of the aging population are. Leisure walking is the focus of this paper, as it is the most prevalent and preferred form of physical activity of older adults [14,15].

The article is organized as follows: The second section will discuss the existing literature on physical activity. Next, we present the data collection. This is followed by descriptions of sample characteristics and walking location choices, and the results of the multinomial logit model estimation. The paper ends with a discussion of the results, conclusion, and limitations.

## 2. Literature Review

### 2.1. Theoretical Foundation

A social-ecological framework served as the theoretical foundation of this study. The social-ecological framework is concerned with people’s transactions with their sociocultural and physical environments [16,17]. A core assumption of the social-ecological framework is that individual behavior is affected by multiple levels of factors. According to McLeroy et al. [18], five levels of factors influence how or why a person engage or fail to engage in a healthy behavior such as physical activity: intrapersonal factors (e.g., gender, age, income), interpersonal factors (e.g., social capital), institutional factors (e.g., access to the workplace), community factors (e.g., neighborhood environment) and public policy (e.g., local, state, and national laws and policies). The socio-ecological framework has been widely used in physical activity studies in the public health and leisure research field [6,19]. This study considers personal and physical environmental factors potentially associated with the location choice of older adults for leisure walking.

In addition, the activity-travel scheduling process includes a number of different aspects such as activity type choices, start time choices, time expenditure choices, activity location choices, travel mode choices, etc. They are recognized as interrelated as a network. For example, Hägerstrand [20] argued that individuals are bounded in time and space when implementing their daily activities and some places are accessible only at designated times. Rasouli and Timmermans [21] contended that the same activity of longer duration might be conducted elsewhere so as to save travel time in the case that the available time window for conducting an activity becomes too small. Habib and Hui [22] found that older people’s activity type and location choices are more closely coupled during the latter part of the day than during the earlier part of the day. Therefore, this study also considers the start time, duration of leisure-time walking and the considerations of later activities in a chained trip which are potentially associated with the location choice for leisure-time walking as a specific type of activity behavior.

### 2.2. Individual-Level Characteristics and Physical Activity

A number of socio-demographic variables have been explored that have a significant influence on physical activity of older adults such as gender, age, income, educational level and household structure, such as presence of grandchildren. For example, Janke et al. [23] and Weiss et al. [24] found that older males are more actively engaged [23] in physical activity than older females. Milanovic et al. [25] examined the differences in physical activity levels between young and old elderly in Serbia and concluded that the reduction in physical activity levels is due to the aging process. Moschny et al. [26] analyzed barriers to physical activity in older adults in Germany. They found that lack of opportunities for sports or leisure activities was more frequently stated by female respondents than male respondents and poor health was more frequently considered a barrier to physical activity by participants aged 80+ years compared to the younger age group. By revealing differences between men and women, and age groups, they concluded that physical activity intervention strategies should be tailored to the specific subgroup of older adults to reduce their corresponding constraints to physical activity. Janke et al. [23] and Weiss et al. [24] found that physical activity participation is lower among less educated elders. For the relationship between education and walking, Clark & Scott [27] believed that the reason could be that more educated people have a better understanding of the benefits of walking than those with lower education levels and thus are more likely to walk. Low income has also been found to be significantly associated with less overall walking levels among older adults [28]. Feng [29] found that the elderly living in the household with grandchildren tend to travel short distances than seniors who live alone or as couples.

In addition to the abovementioned factors, large heterogeneity among seniors in physical activity is also observed, varying by skills, self-efficacy, social support and intentions. For example, Yi et al. [30] found that physical activity skills are significantly associated with older adults’ participation in physical activity. However, skills will not be taken into account in this study, as walking does not require any special skills. Self-efficacy refers to people’s belief in their capabilities to perform a behavior [31]. Pan et al. [32] found that higher self-efficacy is associated with higher odds of reporting sufficient physical activity for older adults. In our study, we used older adults’ real walking duration on a trip as a proxy of self-efficacy and estimated its relationships with the choice behavior of walking locations. Orsega-Smith et al. [33] found that social support is significantly related to leisure-time physical activity of older adults. Many studies have demonstrated that a general factor of social support underlies the different dimensions of supportive behaviors such as instrumental, information, emotional, appraisal and companionship support [34,35]. Oka et al. [36] concluded that social support specific to exercise is an even better predictor than general social support measures. For the purpose of the present study, social support specifically refers to companionship support in this paper. According to the theory of planned behavior, personal intention is a determinant of the implementation of behavior and the stronger a person’s intention is to undertake a behavior, the more likely the behavior will be performed [37]. However, an individual’s intention to undertake a certain behavior grows stronger in line with increased social support or heightened self-efficacy in controlling a situation [38]. As we have considered social support and self-efficacy, we will not consider intentions in this study.

Overall, these studies suggest that heterogeneity exists among older adults in physical activity behavior and intervention strategies should be tailored. For example, for less educated older people, physical activity interventions might better target educating them on the health benefits of physical activity; for older adults who are more likely to be affected by barriers to physical activity, physical activity interventions might better target reducing barriers. However, these studies do not give insight into whether heterogeneity in choice behavior of physical activity locations among older adults exists as a function of individual-level characteristics.

### 2.3. Neighborhood Characteristics and Physical Activity

Apart from individual-level characteristics, neighborhood characteristics also play an important role in shaping an individual’s physical activity. As the neighborhood environment can be modified, identifying specific modifiable environmental factors that can encourage physical activity has been the focus of many scholars in the field of public health, leisure research, urban and transportation planning, in order to help support the idea that these modifications to the environment are worth the investment. A growing body of research has documented the relationships between neighborhood environment and physical activity. For example, Inoue et al. [39] examined the relationship between perceived neighborhood environment and walking for specific purposes among Japanese older adults. Results indicated that access to shops is strongly positively associated with total weekly walking time for transport by older women. Van Cauwenberg et al. [40] systematically reviewed the research into the relationships between environmental attributes and leisure time physical activity of older adults. They found no evidence of significant relationships with access to shops and strong evidence of a positive association between an aesthetically pleasing scenery and leisure time walking of older adults. Cerin et al. [41] concluded that the amount of recreational walking in Hong Kong older adults is positively related to the availability of parks.

Despite substantial research examining physical activity in relationship to neighborhood characteristics, these studies have yielded inconsistent results [5,42]. One possible explanation for this inconsistency is the use of context-free measures of physical activity, such as total amount of physical activity or walking [43,44]. Specifically, mismatch between where physical activity or walking takes place and where environmental attributes are measured may result in null findings.

To date, an increasing volume of research has used context-specific physical activity measures to understand their relationships with environmental attributes. One line of the research focuses on examining the relationships between public open space attributes and physical activity occurring in public open spaces. For example, Sugiyama and Ward Thompson [45] examined what aspects of neighborhood open space are associated with walking for recreation. They found that the pleasant open space items relevant to the suitability for chatting with people and children’s play, and the quality of trees and plants affect the level of use of these places for recreational walking. Sugiyama et al. [46] examined associations of attractiveness, size, and proximity of multiple neighborhood open spaces with recreational walking. They concluded that the presence of a large and high-quality park within walking distance of one’s home may be more important than the presence of open space within a shorter distance. Similarly, Giles-Corti et al. [47] found that simply providing proximate public open space is insufficient to attract walking. Koohsari et al. [43] concluded that close proximity is less important for walking than attractiveness and quality of the open spaces.

Besides the focus on the attributes of green open spaces as destinations, other researchers highlight the influence of route attributes and overall characteristics of the neighborhood environment such as footpaths, traffic safety and aesthetics on the use of green open spaces for physical activity including leisure-time walking. For example, Kaczynski et al. [48] examined the association between road traffic speed and neighborhood residents’ park-based physical activity. They concluded that safe access to parks through traffic speed reduction strategies could facilitate park-based physical activity. Koohsari et al. [43] examined how perceptions of the surrounding built environment are associated with green open space-related walking. They concluded that safety from traffic and aesthetics is associated with greater use of green open spaces for walking. Sugiyama and Ward Thompson [45] found that the condition of footpaths is relevant to older people’s use of green open spaces.

Another line of research focuses on examining the relationships between neighborhood or street characteristics and street-based physical activity. For example, Koh and Wong [49] assessed which infrastructural compatibility factors affect people’s walking route choice and found that the probability of selecting the route is higher if it has better scenery and more shops. Sugiyama et al. [50] reviewed articles to examine the correlates of recreational walking. They found that one-fifth of the relevant studies concluded that areas with utilitarian destinations (i.e., local shops) may attract recreational walkers. However, Sung et al. [51] found that better accessibility to parks plays a role in decreasing substantive walking activity on the streets even with shops. It seems that the presence of shops can only affect the choice among streets but not the choice between the street and the park.

Overall, in recent research on physical activity, an increasing interest has been paid to examine what environmental characteristics are associated with the specific use of neighborhood streets or parks for physical activity. Such research has overcome the limitation of research on context-free physical activity. However, systematic research examining the interactions between neighborhood characteristics and physical activity locations types is limited. In other words, it is unclear whether the importance of neighborhood characteristics might differ for the use of parks and the use of streets or others for physical activity. Therefore, it is challenging to prioritize appropriate environmental interventions.

### 2.4. Summary and Focus of Current Research

In summary, leisure research and public health scholars have attempted to examine the relationships between individual-level characteristics, neighborhood characteristics and physical activity or walking of older adults. There is a general consensus that these characteristics play an important role in shaping older adults’ physical activity or walking behavior. However, fewer investigations have examined the effects of contextual features of activity participation, socio-demographics and neighborhood characteristics on location choice for leisure-time walking of the aging population. Such analyses could have implications for policy makers to determine which location types should be given a priority for intervention to promote leisure-time walking and what environmental attributes should be intervened to better support the use of a specific location for leisure-time walking or maximize the use of different locations to benefit the largest number of people. Given these gaps, the present research sought to address the following research questions:

(1) Are there differences in the choice behavior of leisure-time walking locations of older adults due to the effects of socio-demographic characteristics?
(2) How do contextual features of activity participation such as activity time and trip purpose influence the choice of locations for leisure-time walking?
(3) Do neighborhood characteristics play different roles in the use of different locations for leisure-time walking?

## 3. Materials and Methods

### 3.1. Data Collection

We choose the Dalian urban area as our study area to examine the location choice for leisure-time walking of the elderly in the Chinese context. Located in one of the three largest coastal urban agglomerations with the most competitive economies in China, Dalian is a high-density, mixed-use city with a built-up area of 396 km^2^ and a population of 3.05 million [52], which is different from many low-density cities in Western countries. The area of park green space and neighborhood green space were 30.4 km^2^ and 20.4 km^2^ respectively [53]. In the three agglomerations, there are many cities with similar urbanization levels and urban (neighborhood) characteristics to Dalian [54]. Thus the research findings in Dalian might be typical and informative of the type of cities.

In this study, we use a data set that was collected in a larger study involving a 7-day outdoor routine activity recall. The data were collected in diverse neighborhoods in Dalian, China. The neighborhoods were purposively selected from three location categories, namely, the inner city, the fringe of the city and the area between the inner city and the fringe, in order to have substantial variations with respect to the neighborhood environmental characteristics. Also, considering the fact that different older people might have different preferences for outdoor activity locations and inclusion of such diverse older people is important to reduce sampling bias, participants were recruited from different outdoor locations such as streets, local squares and parks, etc. Finally, 391 surveys were completed between August and September 2017, out of which 28 were eliminated due to missing information or inaccurate records, etc. All subjects gave their informed consent for inclusion before they participated in the study. The study was conducted in accordance with the Declaration of Helsinki. The questions were asked in Chinese but were translated for the purpose of this manuscript.

### 3.2. Measures

#### 3.2.1. Socio-Demographics

The following socio-demographic variables were collected: gender, age, income level, and household composition. In addition, each participant’s functional capability was assessed. They were asked to respond on a scale (ranging from not at all; a little; a moderate amount; very much; an extreme amount), to the following question: “To what extent has your functional capability hindered you from engaging in routine outdoor activities”.

#### 3.2.2. Neighborhood Characteristics

Variables related to the neighborhood characteristics included: accessibility to local shops, footpath conditions, neighborhood aesthetics, and safety from traffic. To measure accessibility to local shops, respondents were asked to report their perceived distance to their most known or frequently visited shopping place using the duration in minutes. Regarding footpath conditions, neighborhood aesthetics and traffic safety, respondents were asked to indicate their extent of satisfaction with each one on a five point Likert scale. Distance to the nearest park was objectively measured using ArcGIS 10.4 (Environmental Systems Research Institute, Redlands, CA, USA) combined with Baidu Map, and the distance measure was taken using network distances.

#### 3.2.3. Leisure-Time Walking Behavior

To measure routine outdoor activity behaviors, an interviewer-administered questionnaire involving a 7-day recall was used. Firstly, respondents were asked to select all the habitual activities that are conducted in a typical week. As habitual activities are routinely conducted with some degree of regularity, the recall data will not be severely biased. Secondly, they were promoted to provide detailed information on each activity episode: start time, origin, destination, travel mode, route names or bus number if travelling by bus, trip duration, and activity duration. Then, respondents were asked to indicate whether they always go back home after the activity. If yes, they need to provide the trip information: time, origin, destination, travel mode, route names or bus number if travelling by bus, trip duration; if no, provide the same relevant information on the subsequent activity they performed as the first activity episode and then provide the trip-back-home information until there is no additional activity during a single tour. Thirdly, the frequency of each home-based tour and the day(s) of the week when it is conducted were solicited. Considering the situation that respondents sometimes may conduct the same activity at a different location or start at a different time, etc., the detailed activity-travel information on the same activity type under another spatial-temporal context is also needed to be specified.

The process of developing the sample for this study involved several steps. First, individuals who participate in leisure-time walking in a typical week were selected from the larger group of the population. Second, their weekly leisure-time walking activities and travel episodes were selected from the larger activity survey database. Third, travel episodes that began and ended at home with only leisure-time walking in-between were selected, labelled as solo purpose trips, and those with leisure-time walking and other activities labelled as joint purpose trips. Finally, the socio-demographic and environmental characteristics were appended to each activity episode based on the ID number of the respondents. The final sample for analysis includes 3184 weekly leisure-time walking activity episodes of 316 individuals.

### 3.3. Data Analysis

Descriptive analyses were conducted for all independent and dependent variables. Relationships between independent and dependent variables were estimated using the multinomial logit (MNL) model [55]. The MNL was selected in our study because the dependent variable was categorical and unordered. In diverse fields such as marketing, transportation, health and urban economics, the most widely used method to model choice among mutually exclusive alternatives has been the MNL [56,57]. The underlying theory of the MNL posits that individuals choose the option of maximal benefit or utility when confronted with a discrete set of options. The utility of a choice relative to the reference category is assumed to be a linear function of the characteristics of the possible choices, the characteristics of the person making the choice and the context in which the decision is being made. In our study, the location category−street serves as the reference category. The independent variables aiming to explain location type choice include socio-demographic variables, characteristics of the neighborhood environment, and episode participation occasion variables. The independent variables were effect-coded. This means that for a variable with L categories, L-1 indicator variables are created. Each category corresponds with a value of 1 on the corresponding indicator variables and a value of zero on all other indicator variables. The reference category is coded as -1 on all indicator variables. Consequently, all estimated coefficients sum to zero and estimated parameters can be interpreted as deviations from the mean. The model was estimated using the Econometric software NLOGIT version 5.0 (Econometric Software, Inc., NY, USA).

## 4. Results

### 4.1. Sample Characteristics

The basic socio-demographic characteristics are presented in Table 1. The results show that the sample is almost equally divided by gender.

Age was categorized into four categories. Their frequency distribution in the sample is 21.5%, 19.9%, 20.9% and 37.7%, respectively. Regarding individual income level, the results indicate that middle-income individuals dominate the group. 40.8% of the respondents have physical limitations. Households with no grandchildren make up 83.2% of the sample, while 16.8% lives with grandchildren.

With respect to the characteristics of the neighborhood environments, Table 1 shows that the majority of respondents (73.1%) perceive accessibility to local shops within 10 min, whereas 26.9% perceive it as over 10 min. 26.3% of the respondents live within an 800 m street network distance to the nearest park, 44.9% within an 800–1600 m buffer and 28.8% live over 1600 m away from the nearest park. 14.3% of the respondents are less satisfied with footpath conditions. 52.2% and 51.9% are less satisfied with neighborhood aesthetics and traffic safety, respectively.

### 4.2. Description of Walking Location Choices

Table 2 shows the distribution of leisure-time walking across four different location types. Most frequently, respondents engaged in leisure-time walking on neighborhood streets. This is consistent with previous studies which showed that neighborhood streets are the setting most frequently used for recreational physical activity, typically for walking [11]. Other common spaces for leisure-time walking included: local squares (27.6%) and parks (21.0%). Only 6.6% of the leisure-time walking takes place in a school playground. This might be related to place attachment or the time of availability.

### 4.3. Model Results

The estimated MNL model results in a McFadden pseudo Rho-squared of 0.247, indicating a good model fit. The estimated coefficients are shown in Table 3. In the following sections, we discuss the effect of variables by category.

#### 4.3.1. Socio-Demographics

Among the socio-demographic variables, the effect of gender indicates that older females are less likely to conduct leisure-time walking in a school playground, in a local square and in a park than males. This result may be explained by the fact that women tend to have more obligations with regard to household duties and are often contracted to more immediate surroundings such as neighborhood streets. Preferences differ between age groups. Estimated coefficients indicate that the 65–69 age group has lower preferences for local squares and parks, and the oldest group is less likely to engage in leisure-time walking in a school playground. It is interesting to find a positive effect on participation in leisure-time walking in a park for the 70–74 age group. This can probably be explained by the fact that this age group has more free time and more outdoor leisure-time pursuits compared to the younger age group, and better physical mobility than the oldest age group, and thus they prefer to conduct leisure-time walking in a park and are able to achieve it. Results for income level indicate that respondents from the low-level income group have a lower tendency to perform leisure-time walking in a school playground and in a park, whereas the opposite holds for the high-level income group. It is quite likely that this result is associated with self-esteem. Low-income seniors tend to have lower self-esteem and thus they prefer their familiar surroundings most for leisure-time walking. The effect of mobility disability shows that older people with mobility disabilities are less likely to participate in leisure-time walking in a park. This result is quite intuitive, since older adults with mobility disability are more sensitive to distance.

#### 4.3.2. Episode Participation Occasion Variables

The time of day of participation in leisure-time walking is an important determinant of the choice of location types. Specifically, individuals are less likely to participate in playground-based walking in the morning, whereas they are more likely to participate in park-based walking in the same period. In the afternoon, they are less likely to perform leisure-time walking in a local square and in a park. In the evening, they are more likely to go to a school playground or a local square for leisure-time walking.

The coefficient of trip purpose in a tour indicates that individuals are less likely to choose a school playground, while they are more likely to choose a local square or a park, if they have other trip purposes (i.e., sitting and relaxing or chatting, dancing, etc.) after leisure-time walking in a tour. This result is quite reasonable, since the squares and parks generally have greater potential to offer the public opportunities for various leisure activities, compared to school playgrounds and streets.

The activity duration variables indicate a lower likelihood to go to a school playground when the respondents want to undertake leisure-time walking for 10–30 min. In addition, the need for long-duration walking (over 60 min) has a positive effect on the choice of playgrounds and squares. However, the result shows a negative effect on the choice of parks. The effect of activity companion shows that individuals with a companion are more likely to conduct leisure-time walking in a school playground and in a park.

#### 4.3.3. Neighborhood Characteristics

The effect of accessibility to local shops shows that individuals who perceive accessibility to local shops as over 10 min are less likely to perform leisure-time walking in a school playground, maybe because they generally count their long-duration walking for shopping as exercise and after the activity they are more interested in places mainly for leisure activities (i.e., park) than places mainly for exercises (i.e., playground). However, the effects of accessibility to local shops on the choice of local squares and parks are not significant. The effect of distance to the nearest park indicates that longer distance to the nearest park is related to a lower propensity for participation in leisure-time walking in a park. A similar relationship is found with participation in leisure-time walking in a local square.

Results for footpath conditions indicate that individuals who are satisfied with footpath conditions are more likely to participate in leisure-time walking in a park. However, these people are less likely to conduct leisure-time walking in a local square, implying a park is normally more attractive than a local square, if there are high quality footpath connections. The coefficient of neighborhood aesthetics indicates that individuals who are satisfied with neighborhood aesthetics are less likely to engage in leisure-time walking in a park. The estimated parameters for traffic safety show that being satisfied with traffic safety has a positive effect on participation in leisure-time walking in a park.

## 5. Discussion

This study analyzed the influence of socio-demographics, episode participation variables and neighborhood characteristics on the location-type choice of older adults for leisure-time walking. A multinomial logit model was estimated based on one-week data collected among 316 respondents aged 60 or over in 2017 in Dalian, China. In contrast to previous studies that mainly focused on the use of a specific type of location for leisure-time walking, the findings of this research contribute toward an understanding of which location types are chosen by which groups of individuals under which conditions.

The results of this study indicate that older women are likely to prefer neighborhood streets as a walking location. The 65–69 age group has lower preferences for local squares and parks, and the oldest group (75+) is less likely to engage in leisure-time walking in a school playground; on the other hand, the 70–74 age group prefers parks more than any other location types. Individuals from the low-level income group have a lower tendency to perform leisure-time walking in a school playground and in a park. Older people with mobility disabilities are less likely to participate in leisure-time walking in a local square and in a park. Overall, it seems that using certain outdoor locations requires older adults to possess certain abilities (e.g., high physical functioning) and other resources (e.g., time, money). Therefore, exploring how the location choices for leisure walking may differ across socio-demographic groups is imperative.

Our study also found that individuals prefer to participate in leisure-time walking in a park in the morning, while they have a high propensity to go to a school playground or a local square for leisure-time walking in the evening. It is likely that the walking locations vary within an individual depending on the timing of activity. Thus, connecting different walking location types into a more integrated system is crucial for meeting the diverse needs of older adults. For example, school playgrounds need to be considered as an integral part of the urban open spaces and should be considered with other types of open spaces simultaneously in the management of urban open spaces, instead of being simply managed by schools to exclude certain groups and make places safer for their own users. Local squares and parks are more likely to be utilized by individuals who tend to chain other leisure activities with walking into a tour. The need for long-duration walking (over 60 min) has a positive effect on the choice of playgrounds and squares, whereas the opposite holds for park use. Individuals with a companion are more likely to conduct leisure-time walking in a school playground and in a park. This might imply that people are more willing to go to a place (i.e., park) which is developed with more attractive qualities and supporting amenities, even though it is relatively further away from their home, if they have a companion to walk with.

Distance from a park is significantly negatively associated with park use. Neighborhood characteristics related to footpaths, aesthetics and traffic are not significantly associated with the use of school playgrounds for leisure-time walking. However, using parks is positively associated with footpath conditions. This is in line with previous studies that found significant relationships between park use and footpath conditions [51,58]. High satisfaction with traffic safety is also positively associated with park use. This is consistent with the work of others who found that safety from traffic is often reported as a barrier to park use [48,59]. In addition, we found that neighborhood aesthetics are negatively associated with the use of parks. However, several previous studies that examined the relationship between neighborhood aesthetics and leisure walking reported different results, i.e., neighborhood aesthetics were positively associated with leisure walking [39,60]. One possible explanation for this inconsistency about the effect of neighborhood aesthetic is that many previous studies used context-free leisure walking measures, which can lead to complicated associations between aesthetics and leisure walking. For example, patterns of associations may differ by context-specific leisure walking (e.g., park-based walking and street-based leisure walking). Future studies are needed to examine whether the effects of neighborhood aesthetics on leisure walking occurring in different contexts are different.

The research results have important implications for the planning, design and management of outdoor open spaces to enhance the efficiency of resource use and the health benefits for older adults. First, the study demonstrates the diversity in older people’s choice for walking location types depending on gender, age, and income level and mobility disability. Consequently, according to local socio-demographic characteristics, urban planners and designers could better target and prioritize appropriate environmental interventions to maintain and promote local older people’s walking while making much needed financial savings. For example, for a neighborhood inhabited mainly by older women, the improvement of the quality of neighborhood streets should be given a priority, as the research shows older females prefer streets for leisure-time walking.

Second, our research suggests that individuals who tend to chain other leisure activities with walking into a tour prefer parks and squares more than any other location type. As the number of older adults increases and they usually have relatively more discretionary time, the size of the group preferring multipurpose trips is likely to become larger, leading to more demands for places such as parks and squares at which multiple leisure activities could take place. Thus, putting much effort into the provision of parks and squares might need to be a goal of urban planners and designers in the future. Furthermore, if it is not feasible to build a park in an area, the provision of squares appears to be a suitable alternative, as both of them have the potential to accommodate the desires for multiple leisure activities including walking at a single location.

Third, this study reveals that preference for parks also depends on distance, footpath conditions and traffic safety. Specifically, our research suggests that a maximum distance of 800 m is recommended as an appropriate walking distance to neighborhood parks. Further, improving older people’s satisfaction with footpath conditions and safety from traffic could encourage them to actively participate in leisure-time walking in the park.

Fourth, our empirical analysis indicates that neighborhood aesthetics play a different role in the choice of streets and parks. Specifically, neighborhood aesthetics could attract older people to perform leisure-time walking on the streets but detract from their use of the park. This is useful for environmental interventions aimed at promoting leisure-time walking because, in practice, the socioeconomic disparities in park accessibility and the disparities in the provision of parks are hard to avoid. Areas lacking access to parks might need to promote older adults to participate in leisure-time walking on the streets through the improvement of neighborhood aesthetics.

This study had several limitations that should be noted. Firstly, data were collected in a specific city of China, potentially limiting the generalizability of our results to other cultural settings. Secondly, we focused on older adults’ leisure-time walking in a typical week. If data had been collected throughout the year, the results might be different, as it might have introduced additional variability in location choice for leisure-time walking across different seasons and weather conditions. Thirdly, the measures of walking behavior relied on self-reports which are often subject to recall bias. Finally, several single items that we used to measure Chinese older adults’ perceived neighborhood characteristics have not been validated. While not expected to be as valid as validated instruments (e.g., Neighborhood Environment Walkability Scale with 68 items), we believe these brief questions are desirable in order to reduce respondent burden and thereby increase response rates, especially in the case where we have had an extensive questionnaire on outdoor activity. Future work is needed to develop and validate a brief questionnaire of perceived neighborhood characteristics related to outdoor activities appropriate for Chinese and other Asian older adults.

## 6. Conclusions

This study demonstrated that contextual features of activity participation, socio-demographics and neighborhood characteristics had significant impacts on the location choice for leisure-time walking of the aging population. The association between socio-demographics and location choice suggested that different socio-demographic groups might prefer different locations for participation in leisure-time walking. Moreover, the results showed that older adults tend to choose different locations for leisure-time walking in different contexts of walking participation. In addition, we found that neighborhood characteristics played different roles in influencing the choice of different types of locations.

Overall, results of this study could inform efforts to promote leisure-time walking and have important implications for policy makers to evaluate the cost effectiveness of creating and improving different locations for supporting leisure-time walking of older adults. It also provides guidance to the management of open spaces on how to develop a partnership between different outdoor location types to realize their synergistic potential for leisure-time walking. Additionally, these findings contribute to find out alternative solutions to sustain older adults’ leisure walking to overcome dilemmas faced by some neighborhoods or cities (i.e., some neighborhoods have parking as the predominant street level use, limiting the opportunities for street-based leisure walking; good quality parks may not be present in sufficient numbers in cities to be accessible to all people).

## Figures and Tables

**Table 1:** Sample characteristics (*n* = 316).

Variable	Levels	*n*	%
Socio-demographics			
Gender	Male	164	51.9
	Female	152	48.1
Age	60–64	68	21.5
	65–69	63	19.9
	70–74	66	20.9
	75+	119	37.7
Income level	0 ≤ 2000 Chinese Yuan	71	22.5
	2001 ≤ 4000 Chinese Yuan	151	47.8
	4000+ Chinese Yuan	94	29.7
Mobility disability	No	187	59.2
	Yes	129	40.8
Household type	With grandchildren ≤ 12 years	53	16.8
	No grandchildren ≤ 12 years	263	83.2
Environmental characteristics			
Accessibility to local shops	0–10 min	231	73.1
	Over 10 min	85	26.9
Distance to the nearest park	Less than 800 m	83	26.3
	800 to 1600 m	142	44.9
	More than 1600 m	91	28.8
Footpath conditions	Less satisfied	45	14.3
	(Very) satisfied	271	85.7
Neighborhood aesthetics	Less satisfied	165	52.2
	(Very) satisfied	151	47.8
Traffic safety	Less satisfied	164	51.9
	(Very) satisfied	152	45.1

**Table 2:** Distribution of activity locations across the four categories.

Activity Locations	*n*	%
Street	1427	44.8
Square	880	27.6
Park	669	21.0
Playground	208	6.6
Total	3184	100

**Table 3:** MNL model for location-type choice for leisure-time walking.

Variables	Coefficient		
	Playground	Square	Park
Constant	−2.300 ***	−0.532 ***	−1.180 ***
Socio-demographics			
Gender			
Male	0.319 ***	0.209 ***	0.528 ***
Female	−0.319	−0.209	−0.528
Age			
60–64	0.628	0.199	0.024
65–69	0.045	−0.278 ***	−0.268 **
70–74	−0.149	0.101	0.191 **
75+	−0.524 ***	−0.022	0.053
Income level			
0 ≤ 2000 Chinese Yuan	−0.167	0.177 **	−0.257 ***
2001 ≤ 4000 Chinese Yuan	−0.175	−0.158	−0.019
4000+ Chinese Yuan	0.342 ***	−0.019	0.276 ***
Mobility disability			
Yes	0.048	−0.191 ***	−0.272 ***
No	−0.048	0.191	0.272
Household type			
Presence of children (≤12 years)	0.062	−0.117	0.052
Non-presence of children (≤12 years)	−0.062	0.117	−0.052
Episode participation occasion variables			
Activity time			
Morning	−0.666 ***	0.017	0.222 ***
Afternoon	−0.008	−0.283 ***	−0.217 ***
Evening	0.674	0.266	0.005
Trip purpose			
Joint	−0.722 ***	0.480 ***	0.315 ***
Single	0.722	−0.480	−0.315
Activity duration			
10–30 min	−0.922 ***	−0.061	−0.113
31–60 min	0.453	−0.221	0.427
60+ min	0.469 ***	0.282 ***	−0.314 ***
Activity companion			
Yes	0.438 ***	−0.062	0.318 ***
No	−0.438	0.062	−0.318
Environmental characteristics			
Accessibility to local shops			
0–10 min	0.337	−0.023	0.075
Over 10 min	−0.337 ***	0.023	−0.075
Distance to the nearest park			
≤800 m (base)	−0.345	0.397	1.071
800–1600 m	0.199	−0.117 *	−0.094
1600+ meters	0.146	−0.280 ***	−0.977 ***
Footpath conditions			
Less satisfied	0.176	0.157	−0.191
(Very) satisfied	−0.176	−0.157 **	0.191 **
Neighborhood aesthetics			
Less satisfied	0.099	0.076	0.387
(Very) satisfied	−0.099	−0.076	−0.387 ***
Traffic safety			
Less satisfied	−0.146	−0.005	−0.185
(Very) satisfied	0.146	0.005	0.185 ***
Goodness of fit statistics			
LL(0)	−4413.96		
LL(β)	−3320.66		
McFadden Rho-squared	0.247		

***, **, * mean significant at 1%, 5% and 10% level.
